# Beliefs About e-Cigarette Regulation Among a Cohort of Long-Term Adult Users: A Thematic Analysis

**DOI:** 10.1177/1179173X261476227

**Published:** 2026-08-20

**Authors:** Gail C. D’Souza-Rushton, Candace R. Bordner, Sophia I. Allen, Nicolle M. Krebs, Andrea Hobkirk, Jonathan Foulds, Jessica M. Yingst

**Affiliations:** 1 Department of Public Health Sciences, Penn State Center for Research on Tobacco and Health, 12310 Penn State College of Medicine, Hershey Medical Center, Hershey, PA, USA; 2 Department of Psychiatry and Behavioral Health, 12310 Penn State College of Medicine, Hershey, PA, USA

**Keywords:** e-cigarette use, FDA regulation, smoking cessation, qualitative research

## Abstract

**Introduction:**

The United States Food and Drug Administration (FDA) is a regulatory authority with the ability to regulate tobacco products, including electronic cigarettes (e-cigarettes). Few studies have investigated the beliefs and attitudes about e-cigarette regulation, from the perspective of long-term users of e-cigarettes. The purpose of the current study was to understand the beliefs about e-cigarette regulation among former and current users of e-cigarettes using a qualitative approach.

**Methods:**

We conducted a cross-sectional survey among a cohort of former and current users of e-cigarettes in 2022 which contained open-ended questions about perceptions of e-cigarette regulation. Participants were asked about their e-cigarette use, whether the FDA should regulate e-cigarettes (yes/no), and responded to an open-ended question about their opinions of FDA regulation/non-regulation of e-cigarettes. Means and frequencies were used to describe the sample, and all open-ended responses were imported into MAXQDA software. Open-ended responses were analyzed using thematic analysis.

**Results:**

Participants (n=172) were 93.0% White, 66.3% male, and 73.3% were people who currently use e-cigarettes. More than half (51.7%) of participants reported they do not believe the FDA should regulate e-cigarettes, while 48.3% of participants reported they believe that the FDA should regulate e-cigarettes. Opponents of e-cigarette regulation felt it would lead to over-regulation and politicization. On the other hand, those with supportive views felt that FDA regulation would ensure the safety of e-cigarette products.

**Conclusion:**

Our findings suggest that this cohort of long -term users of e-cigarettes believe FDA regulation will affect the availability of e-cigarette products, making it harder to quit smoking or to avoid relapsing to traditional cigarettes. User perceptions highlighted that the FDA should assess the health impact of e-cigarette products and their influence on smoking cessation efforts among smokers who had otherwise been unable to quit.

## Introduction

Electronic nicotine delivery systems, commonly referred to as electronic cigarettes (e-cigarettes), are a diverse class of electronic products which heat a nicotine-containing liquid into an aerosol for inhalation by the user.^1^ Since the introduction of e-cigarettes to the market, the types of e-cigarette devices and the characteristics of the products have evolved rapidly.^2^ Regulation of these products is rooted in the Family Smoking Prevention and Tobacco Control Act of 2009, which granted the United States (US) Food and Drug Administration (FDA) authority to regulate the manufacture, marketing and distribution of tobacco products to protect public health. Initially, this regulation applied to cigarettes, cigarette tobacco, and smokeless tobacco products. In August 2016, the FDA’s Rule extended its regulatory authority of tobacco products to include e-cigarettes and e-liquids.^3^ This regulatory authority covers all parts related to e-cigarette use, including e-cigarette batteries, e-liquids, chargers and flavorings. This Deeming Rule was implemented in response to the rapid growth of the e-cigarette market and concerns regarding potential public health harms such as youth vaping and nicotine dependence, exposure to nicotine and potentially other harmful aerosol contents and uncertainty surrounding long-term health effects of e-cigarette use.

Since gaining regulatory authority, the FDA has taken several actions about e-cigarettes such as retailer and manufacturing checks on retailers that make or modify e-cigarettes or e-liquids, increasing regulatory requirements for the manufacturing of e-cigarettes, and creating and enforcing pre-market authorization requirements.^4^ In 2017, the FDA created the Youth Tobacco Prevention Plan which aimed to decrease youth access to e-cigarettes, decrease marketing of tobacco products to youth, and educate youth and their families about the dangers of tobacco products.^5^ In 2020, the FDA restricted the sale of unauthorized flavored cartridges other than those with tobacco or menthol flavors.^6^ This policy was based on evidence indicating that adolescents and young people show a strong preference for flavored e-cigarettes other than tobacco and menthol flavors.^6^ While disposable e-cigarettes were available prior to the 2020 flavor ban in cartridges, the industry dramatically increased their marketing and sales of flavored disposable e-cigarettes after the 2020 flavored cartridge ban.^6^ Consequently, the US market share of disposable (versus cartridge-based) e-cigarettes increased from 26% before the legislation to around 60% after the legislation, with the majority of unit sales for e-cigarettes being flavors other than tobacco or menthol.^7^

E-cigarette manufacturers, and manufacturers of other tobacco products covered by the Tobacco Control Act that were not on the market by 2007, were initially given a deadline of September 9, 2020, to submit an application to market their products, known as a Pre-Market Tobacco Product Application (or PMTA).^8^ It was initially expected that products that had not been granted a PMTA by September 2021 would have to be withdrawn from the market. However, as FDA received millions of PMTA applications, it announced there would be enforcement discretion, such that products that had submitted a timely PMTA application would be allowed to remain on the market, pending a final decision on authorization or denial, while products that had no pending PMTA application would be prioritized for enforcement and removal from the market. As of May 2026, approximately 46 e-cigarette products (6 manufacturers) have been authorized for marketing by FDA, out of over 20 million applications, of which almost 7 million were accepted for review. However, the proliferation of marketing of unauthorized e-cigarette products has continued. For example, as of 2024, it is estimated that over 80% of the almost 300 million e-cigarette unit sales in US convenience stores were unauthorized products.^8,9^ The primary criterion that the FDA must address before authorizing the marketing of a new product is whether the product is “appropriate for the protection of public health?” This requires the FDA to evaluate the risks and benefits of the products to both users and nonusers, including whether the products would increase or decrease the likelihood that current tobacco users would quit or that non-tobacco users would initiate use, as well as the impact on youth tobacco use.^8,10^

Understanding user attitudes and beliefs towards FDA regulation of e-cigarettes is important, as these attitudes and beliefs may contribute to the success of the policies.^11^ For example, low support for e-cigarette regulation suggests that educational campaigns stressing the importance of regulation may be needed to increase compliance.^12^ A study conducted among current US smokers found that about half believed the FDA should regulate newly deemed tobacco products. Participants were most in favor of regulating nicotine liquids. Stronger quit intention was associated with more favorable attitudes towards FDA regulation.^12^

While it is known that attitudes and beliefs about e-cigarette regulation can impact the effectiveness of new policies, few studies have examined these attitudes and beliefs among former and current users of e-cigarettes. Further, the current literature has focused on responses to multiple-choice questions about beliefs. Rich, qualitative data is needed to better understand the rationale behind participants’ beliefs about regulation. The purpose of the current study is to understand the beliefs about e-cigarette regulation among current and former users of e-cigarettes using a qualitative approach. Data from this study can inform the FDA regarding the direct effects of regulating e-cigarette products among (current or former) people who use e-cigarettes.

## Methods

### Sample

Study participants were current and former users of e-cigarettes from a longitudinal cohort of survey participants followed from 2012 to 2022. Originally these participants were surveyed from 2012 to 2015 using a 158-item survey (wave 1).^13^ Recruitment methods included posting a link on websites like e-cigarette-forum.com, webmd.com and the NJOY website. Participants were asked about their e-cigarette use, device characteristics (type, cost, details of usage), related behaviors, and if they were interested in participating in future research. From the 6493 participants who completed the survey, 1863 (28.7%) were interested in future research. More details about the wave 1 survey have been published elsewhere.^13^

In January 2017 (wave 2), June 2019 (wave 3), and May 2022 (wave 4), the 1863 participants who provided their contact information at wave 1 were re-contacted to complete a follow-up survey.^14-16^ To contact participants for follow-up, email invitations with unique links were forwarded to participants up to 4 times and a text message reminder was sent to those who did not begin the survey or did not complete the survey.

This study focused only on data collected at wave 4 in 2022.^17^ Of the 1863 participants who opted into being re-contacted and provided contact details at the Wave 1 survey ending in 2015, 232 (12.5%) participated in the 2022 wave 4 survey. The survey invite included a link to a description of the survey and a statement that continuing on to the survey implied voluntary consent to participate in the research. This study was approved by the Penn State College of Medicine Institutional Review Board on May 24^th^, 2022 (Protocol # - STUDY20302). The survey data were collected and managed using REDCap electronic data capture tools hosted at the Penn State Milton S. Hershey Medical Center and College of Medicine. REDCap (Research Electronic Data Capture) is a secure, web-based application designed to support data capture for research studies.^18^

### Measures

Demographic information, including age, gender, race, ethnicity, and education level were obtained from participants. Participants were asked to report on their current use of e-cigarettes by answering the following question, “Have you used an electronic cigarette in the past 30 days?” (yes/no). Those who responded ‘yes’ were asked to indicate how many days out of the past 30 have they used e-cigarettes and how long ago did they last use an e-cig.

To inquire about e-cigarette use that would be helpful to researchers for the future, participants were asked the open-ended question, “Please add any additional information you think a public health researcher should know about electronic cigarettes.” In addition, we also asked about participants’ opinion of the Food and Drug Administration (FDA) regulation with the question, “The Food and Drug Administration (FDA) is responsible for protecting the public health by ensuring the safety, efficacy, and security of human and veterinary drugs, biological products, and medical devices; and by ensuring the safety of our nation’s food supply, cosmetics, and products that emit radiation. With this in mind, do you believe that the FDA should regulate e-cigarettes?” Participants who answered yes/no to this question, subsequently received a follow-up open-ended question, “Please tell us why you think this way.” Participants who chose to answer this question typed their answer in free text box. A copy of the complete 2022 survey questionnaire can be viewed in Supplementary Appendix 1.

### Data Analysis

Participants included in the analysis were those who answered “yes” to the question, “Have you used electronic cigarettes on more than 30 days in your lifetime?” and provided a response to the open-ended question about perceptions of FDA regulation. Current and former people who use e-cigarettes were included in the analyses.

Means and frequencies were used to describe the sample. All data, including the open-ended survey responses, were directly entered by participants into the REDCap data collection instrument, then downloaded and imported into MAXQDA software for coding.^19^ Open-ended responses were analyzed using an inductive thematic content analysis approach, with codes and themes developed directly from the data using descriptive orientation. Researchers (CB and GD) independently reviewed the responses and generated codes for all data. The researchers are public health researchers with experience in tobacco and nicotine research and met to discuss the independently developed codes and generated the final codebook. Researchers first coded 20% of the dataset to establish inter-rater reliability while meeting regularly to revise codes, interpretations, and discuss potential influences of their perspectives on the analysis, until a kappa coefficient of >.80 was achieved.^20^ Then, the remainder of the data were then coded by researchers (CB and GD). The six steps of thematic analysis were followed from codebook development, through thematic development.^21^

## Results

### Quantitative Results

232 participants responded to the Wave 4 survey. 198 participants said “yes” to using e-cigarettes for more than 30 days in their lifetime and responded “yes” or “no” to the question inquiring about if they believe the FDA should regulate e-cigarettes. From 198 participants, only 172 participants responded to the open-ended question and were included in the final analysis. Participants (n=172) were a mean age of 52.2 (SD=12.4) years, 66.3% (n=108) male, 93.0% (n=147) White, and 3.1% (n=5) were Hispanic/Latino. All participants used e-cigarettes for more than 30 days in their lifetime, with 126 (73.3%) reporting current e-cigarette use in the past 30 days. Demographic data for participants can be found in Table 1.

**Table 1.** Sample Characteristics of Participants (n=172)

Characteristics	% (n)
Age, mean (SD), years (n=163)	52.2 (12.4)
Male, % (n) (n=161)	66.3 (108)
Race (n=158)	​
Caucasian/White, % (n)	93.0 (147)
African American/Black, % (n)	1.9 (3)
American Indian/Alaskan Native, % (n)	1.3 (2)
Asian, % (n)	0.6 (1)
Other, % (n)	3.1(5)
Hispanic or Latino, % (n) (n=163)	3.1 (5)
Education level (n=161)	​
High school or less, % (n)	8.1 (13)
Some college/technical school, % (n)	34.8 (56)
College degree, % (n)	43.5 (70)
Graduate degree, % (n)	13.7 (22)
Current e-cigarette user (in past 30 days), % (n)	73.3 (126)
Mean times per day usage (SD) (n=125)*	18.7 (18.5)
Ever smoked cigarettes, % (n), (n=167)	97.0 (162)
Currently smoke cigarettes (in past 7 days), % (n), (n=164)	9.8 (16)
Individuals who responded “yes” to regulating e-cigarettes (n=172)	48.3 (83)
Individuals who responded “no” to regulating e-cigarettes (n=172)	51.7 (89)

*Note.* Varying denominators of characteristics denote missing data.

*one “time” consists of around 15 puffs, or lasts around 10 minutes.

### Qualitative Results

#### *Reasons why Users of e-Cigarettes Support FDA regulation of e-Cigarettes*

Four key themes emerged regarding underlying support (n=83) for FDA regulation of e-cigarettes: 1) to ensure access of products to adults only, 2) to ensure purity of ingredients, consistency of nicotine levels, 3) to reduce nicotine dependence, and 4) to regulate manufacturing, not consumer use. These themes were defined as the following: First, participants supported regulations that restricted access of e-cigarettes to youth while preserving access to adults only. Second, participants emphasized the need for purity of ingredients and standardization in levels of nicotine in e-cigarettes to ensure product quality and safety. Third, participants viewed regulation as a method of reducing nicotine dependence and preventing nicotine addiction. Finally, participants highlighted the importance of regulating the manufacturing processes of e-cigarettes but opposed regulations that would unnecessarily restrict adult consumers’ ability to use e-cigarettes.

### Ensure Access of Products to Adults Only

Participants felt strongly about ensuring access of e-cigarette products to adults only and preventing access of e-cigarette products to minors. One participant said, *“Do not want underage (<18) kids to have access to these products. Akin to alcohol and tobacco.”* (Male, W, 64 years old, current e-cigarette user). Another participant reported “yes” to FDA regulation, *“mainly to ensure minors are not using nicotine products”* (Male, W, 42 years old, current e-cigarette user).

### Ensure Purity of Ingredients, Consistency of Nicotine Levels

One participant said “yes” to FDA regulation, *“to make them safer for people, and to ensure ingredients are consistent and as safe as they can be”* (Male, W, 43 years old, current e-cigarette user). Another participant claimed, “*Appropriate regulation of consumer products ensures consistency as opposed to “black market” where the product content is not assured.”* (Male, W, 64 years old, current e-cigarette user). Another participant was in favor of regulation but not regarding regulation of flavors, they said, “*Regulation for ingredient purity and proper contents by volume listed on the label. It should be more weights and measures than banning flavors.”* (Male, W, 48 years old, current e-cigarette user).

### Reduce Nicotine Dependence

In addition to purity of ingredients, some participants were concerned about nicotine addiction from e-cigarettes. One participant highlighted their concern about nicotine addiction by reporting, “*Nicotine is so addictive. I hate that I can’t quit. I wish it was illegal”* (Female, W, 41 years old, current e-cigarette user). Another participant said, *“There should be controls in place to regulate a dangerous chemical like nicotine when used by the general public.”* (Male, W, 36 years old, former e-cigarette user).

### Regulate Manufacturing of e-Cigarettes but Do Not Regulate Consumer Access of e-Cigarettes

Participants were supportive of regulating e-cigarettes to ensure their safety, but they did not support regulations that could impede access to e-cigarettes. One participant said, *“Nicotine is a drug and needs to be regulated. However, the e-cigarette regulations that currently exist, are BS. The FDA is essentially trying to make it so people are not able to purchase ANYTHING related to e-cigarettes.”* (Female, W, 53 years old, current e-cigarette user). Similarly, another participant said FDA should regulate e-cigarettes, *“Only to the extent that manufacturers are producing the products in a manner safe for public consumption. Not to 'regulate' usage. I feel that vaping IS safe as evidenced by my 9.5 years of use to quit smoking and stay smoke free combined with the fact that my doctor has seen, and commented on the actual improvements in my health.”* (Male, W, 57 years old, current e-cigarette user).

#### *Reasons why Users of e-Cigarettes Do Not Support FDA regulation of e-Cigarettes*

Eight key themes emerged regarding discord (n=89) for FDA regulation of e-cigarettes: 1) the focus is not on harm reduction, 2) infringement on e-cigarette user rights, 3) over-regulation of e-cigarettes, 4) other products are more harmful than e-cigarettes, 5) lack of trust in the FDA, 6) limited FDA understanding of real-world e-cigarette use, 7) e-cigarette regulation is related to financial gain, and 8) the FDA is aligned with current political climate and not with current research.

These themes were defined as the following: First, participants expressed concern that FDA regulation of e-cigarettes does not prioritize harm reduction and may limit access to products that could help adults reduce or quit smoking. Participants also viewed regulations as an infringement on individual rights, and that adults should be free to make their own decisions regarding e-cigarette use. Some participants perceived e-cigarettes as being subjected to excessive regulation. Participants also compared e-cigarettes to other harmful substance use products such as alcohol which they considered more harmful to health and questioned why e-cigarettes receive more regulatory attention than alcohol or other products. Some participants expressed lack of trust in the FDA and questioned whether the agency acts in the best interests of people who use e-cigarettes. Participants believed that people who work for the FDA lack sufficient understanding of how e-cigarettes are used in real-world settings, especially with regard to smoking cessation. Participants also suggested that regulatory decisions may be influenced by monetary interests rather than public health considerations of users of e-cigarettes. Finally, participants perceived FDA regulation of e-cigarettes as potentially being influenced by political priorities and the current political climate as opposed to the available scientific evidence of e-cigarettes.

### Focus Is Not on Harm-Reduction

Many participants highlighted the benefits of using e-cigarettes as a method for smoking cessation and harm-reduction. One participant said,

*These products are the most frequently used and most effective stop smoking tool available. If combustible cigarettes really are the most deadly consumer product available, then FDA should be channeling all available resources into encouraging smokers to switch.* (Male, W, 57 years old, current e-cigarette user).

Another participant also reported how she used e-cigarettes to quit smoking. She said,

*There isn't enough room in this box. I quit a 40 year cigarette addiction in 2012 using ecigs. If I was trying to quit now I'm not sure I could, there are so many obstacles to getting products and so much misinformation advertised. No one said vaping is without risk, but smoking is worse. It should be about harm reduction not “protecting the f-ing children.”* (Female, W, 63 years old, current e-cigarette user).

Another e-cigarette user also highlighted the positive effects of using e-cigarettes saying*,*

*Vaping to me is a safer product with great health benefits for users that are already addicted to nicotine. For these groups I am strongly against any kind of regulation that will force us to go back to burning tobacco, which I'm sure is extremely more unhealthy and dangerous. I have experienced a big health improvement after quitting smoking in 2011 and never want to go back again.* (Male, W, 49 years old, current e-cigarette user).

### Regulation Feels Like Infringement on e-Cigarette User Rights

Some participants also reported that they feel the government is infringing on their rights and as adults they should be allowed to control what they put into their body. One participant said, *“I am an adult and can think for myself”* (Female, W, 71 years old, current e-cigarette user). Another participant said the reason they disagree with FDA regulation is,

*Because all they do is screw things up and make a product that can save lives less accessible. I'm an autonomous adult who can decide what my risk tolerance is as well as make my own health decisions.* (Female, W, 36 years old, current e-cigarette user).

### Over-Regulation

Other participants also voiced their concerns regarding over-regulation of e-cigarettes. One participant said,

*It's already governed by consumer protection laws. If it's to be regulated by FDA then it should only be as far as specifications for production (like supplements), not regulated like smoked tobacco, as it is not smoking and nothing is burned.* (Male, W, 53 years old, current e-cigarette user).

Another person mentioned,

*They [FDA] would never get it right and/or will go overboard. Even though quality might improve in theory, the industry is self-regulating (in that people will openly discourage brands that are of poor quality, so they generally strive for best quality)* (Male, W, 28 years old, current e-cigarette user).

### Other Products May Be More Harmful Than e-Cigarettes

Participants also reported that they feel other products such as alcohol, tobacco, fast food, and sugar are more harmful to the public than e-cigarettes and the FDA should be focused on regulating those products versus e-cigarettes. One participant said,

*I think some regulations for ecigs are acceptable. However, the FDA has a track record of banning cool* s**t *because “think of the children,” or more recently, “because menthol is disproportionately marketed to black people.” I'm an adult white male and I think candy and menthol are DELICIOUS. I also realize that tobacco is terrible for me and ecigs probably aren't much better, but so is eating too much sugar, eating fast food at all, and drinking too much alcohol. According to the CDC there are currently ∼30.8 million smokers in the US. More scarily there are 178 million obese people in the US. I think being concerned with ecigs at all is a waste of the FDA's resources. Let's fix the bigger problems and leave the fringe vices alone.* (Male, W, 32 years old, former e-cigarette user).

Another participant said,

*People have rights to choose what they want to use, if they want to take away vape pens then they should get rid of all alcohol because alcohol kills more people and I've never seen someone smoke and hit another person like people who drink beer or vodka and stuff like that which is just as harmful or worse yet they only worry about smokers.* (PID 9285 - Female, W, 41 years old, current e-cigarette user).

### Users of e-Cigarettes Lack of Trust in the FDA

Many participants highlighted that they did not trust the FDA, and were not, “*sure they actually do anything useful”* (Female, W, 42 years old, current e-cigarette user). One participant also said, *“I don’t trust them [FDA] not to protect Big Pharma and Big Tobacco to the detriment of consumers”* (Female, W, 52 years old, current e-cigarette user). Another e-cigarette user felt that the FDA was not qualified enough to regulate e-cigarettes. He said,

*The FDA is not qualified to regulate recreational consumer products. Truthfully the FDA isn't competent to regulate pharmaceutical products (its actual mandate) and any attempt to regulate nicotine products is usurpation of power and is unconstitutional*. (Male, W, 43 years old, former e-cigarette user).

One participant even suggested the creation of another entity to regulate e-cigarettes. They said, *“A new agency specializing in the health benefits and risks of e-cigarette use should be formed to regulate the products.”* (Male, Other - Hispanic, 41 years old, current e-cigarette user).

### Limited FDA Understanding of Real-World e-Cigarette Use

Participants also felt that the FDA does not understand the real-world applications of e-cigarette use and refuse to listen to users regarding e-cigarettes. One participant said he did not want the FDA to regulate e-cigarettes,

*Because the FDA knows next to nothing about it. Vaping isn't like tobacco. The one area that bothers me is flavors (I don't use them.). They are food flavoring, They've never been tested for inhalation. The rest of the juice ingredients are pretty safe.* (Male, W, 65 years old, current e-cigarette user).

Another participant said he feels that, *“The FDA is not willing to actually listen to users to determine how to deliver what they actually want safely”* (Male, W, 54 years old, former e-cigarette user). One participant also highlighted how they aren’t completely opposed to regulation, they said,

*It's not that I'm opposed to regulation, it's just that the current approach seems to rely on a hermeneutic of suspicion that does more harm than good.* (Male, W, 32 years old, former e-cigarette user).

Another participant highlighted their concern about FDA’s approach to e-cigarettes. They expressed that e-cigarettes should not be regulated,

*The former head of the world health organization has stated that vaping may be the single greatest health innovation in the last 100 years. And yet the FDA wants to take the approach that vaping is worse than cigarettes! Over 440,000 people a year die of smoking related illnesses. Not a single person has died from vaping traditional e-liquid.* (Male, W, 53 years old, current e-cigarette user).

### Regulation Is Related to Financial Gain

Some participants felt that FDA regulation is related to financial gain to federal authorities. One participant said, *“In my opinion the banning and strict regulations are strictly a money grab. The government is more concerned with lost tax revenue than individuals health”* (Male, W, 57 years old, current e-cigarette user). Another participant said, *“Federal regulations lead to federal overreach. I agree that some entity “in charge” that actually knew about vaping can help. But not some entity that has financial skin in the game.”* (Male, W, 67 years old, former e-cigarette user). Another participant also believes that, *“FDA regulation often places such a high barrier of entry to the market that only manufacturers with deep pockets are able to enter. Innovation and small business participation is important.”* (Male, W, 44 years old, former e-cigarette user). Finally, one participant also mentioned how tobacco generates more revenue when compared to vaping saying,

*Sadly, here in the US too much money is made from tobacco by the states for them to ever allow vaping to continue. The vape industry cannot generate the amount of revenue that tobacco has! That is the sole drive for trying to eliminate vaping!…The FDA wants to push towards just tobacco if they allow anything at all.* (Male, W, 53 years old, current e-cigarette user).

### FDA Is Aligned With Current Political Climate, Not Current Research

A few participants felt that the FDA was more aligned with the current political climate as opposed to current research about e-cigarettes. One participant said, *“FDA is a political organization. They are not interested in providing an alternative to smoking.”* (Male, W, 66 years old, current e-cigarette user). Another participant said, *“They [FDA] bow to political pressure, and ignore the science.”* (Male, W, 81 years old, current e-cigarette user).

## Discussion

In this study, we examined the beliefs about e-cigarette regulation among current and former adult users (mean age of 52.2 years (SD=12.4)) of e-cigarettes using a qualitative approach. Our findings highlight both public support and discord for the regulation of e-cigarette products. There was a consensus among participants that regulations should ensure access of products to adults but should prevent youth from becoming addicted to e-cigarettes. Although the FDA has restricted the sale of tobacco and tobacco products to age 21 and over, youth are still able to access these products illegally. Nonetheless, youth e-cigarette rates have been dropping over recent years as seen from the 2025 National Youth Tobacco Survey where overall tobacco product use among US high school students declined during 2022-2025 (from 16.5% to 9.7%) with a significant decline driven by e-cigarette use (14.1% to 7.1%).^22^

Participants were also concerned about the accuracy of nicotine levels in e-cigarettes and the need for safer products with consistent/standardized nicotine levels. In the past, there has been a history of unregulated nicotine liquid, with e-cigarette liquid contents and amounts not matching the manufacturing label.^23^ Thus, it is important for consistency to be present in e-cigarettes. Accurately reporting the nicotine concentrations present in a vape product can help users of e-cigarettes monitor their nicotine intake levels and help them reduce their nicotine content over time. A few participants also highlighted the need to regulate the manufacturing of e-cigarettes and not just the product itself. Many users of e-cigarettes from our study reported they agree that parts of an e-cigarette and how it is developed must be regulated, but users of e-cigarettes should have autonomy over what they put in their bodies rather than having the government regulate the product for adults. Finally, participants commented on the use of e-cigarettes as an aid to reducing or quitting smoking and the many health benefits that they see as a result of this. Findings from recent meta-analysis of clinical trials^24^ and observational data collected from national surveys^25^ support this, finding that the use of nicotine e-cigarettes is associated with quitting smoking.

Many participants reported they felt e-cigarettes are already governed by existing laws, and do not need any further regulation. Some participants also reported that the government should have a third party/entity that monitors e-cigarette products since they did not trust the FDA to have the best interest of people who use e-cigarettes in mind. Finally, participants also highlighted that the government should understand real-world applications of e-cigarette use before creating regulations. 

E-cigarettes were linked to the Electronic Cigarette and Vaping-Associated Lung Injury (EVALI) outbreak in 2019 despite later evidence pinpointing Vitamin E acetate-contaminated illegal THC vapes as the cause.^26^ While some case reports continue to report occasional cases of EVALI in people who reported they did not use THC products, the most thorough study found that 48/51 probable and confirmed EVALI patients had Vitamin E Acetate (VEA) detectable in their lungs, and 47/50 had evidence of THC use, while none of the 99 control participants (who included 18 nicotine e-cigarette users) who showed no signs of EVALI had any evidence of VEA in their lungs.^27^ Despite the reporting of occasional new case reports of EVALI-like symptoms in e-cigarette users who deny THC product use, the causes of symptoms in these cases are usually unclear and THC is often unmeasured. In the study, 9/11 EVALI cases who reported no THC use had detectable THC in their body fluids. All of this evidence suggests that both the general public and health professionals over-estimate the risks from nicotine e-cigarettes, partly due to the continuing misperception that nicotine e-cigarettes caused the EVALI outbreak.^28^ Thus, there is a lot of misinformation regarding e-cigarettes amongst the public, which is why users of e-cigarettes feel that the government does not understand the real-world applications of e-cigarette use or does much to correct the misinformation.^29^

Users of e-cigarettes also mentioned they want researchers to conduct *more* research since we are still learning about e-cigarettes and know little about the long-term effects of e-cigarette use. Some participants have found e-cigarettes to be helpful in reducing their harm from cigarettes, and while the goal of FDA regulation is to reduce harm, people who use e-cigarettes feel that restrictions are not in line with reducing harm. Thus, the FDA should continue to evaluate its regulations to ensure that current and future people who use e-cigarettes are able to reduce their health risks by switching to FDA-authorized e-cigarettes. Overall, our findings suggest that e-cig users support regulations that improve product safety, quality and prevent youth vaping initiation, but they are also concerned that excessive regulation may reduce access to e-cigarettes for adults who use them as a harm-reduction or smoking cessation tool.

Limitations were noted in this study. This was a selective sample size of long-term engaged adults who use e-cigarettes, limiting generalizability. Re-contacting the same individuals for the past ten years may have introduced selection and survivorship bias. Researcher reflexivity and potential analytic bias may have been present since the researchers are tobacco prevention experts and their personal opinions on e-cigarettes may have influenced the research. Limited demographic diversity may also limit generalizability of our findings to the US population of people who use e-cigarettes. Sample size/power analysis was not performed for this study. Additionally, the policy and media context at the time of data collection may have influenced participants feelings towards the FDA since data was collected in 2022 (post COVID-19 pandemic) whereby there were a lot of strong feelings in the media toward government entities. The wording of the question inquiring about the opinions of FDA regulation of e-cigarettes by giving participants a relatively detailed description of the FDA may have been a possible source of response priming and potentially shaped responses. Strengths of this study included the collection of rich qualitative data from a large sample of experienced people who use e-cigarettes. Unfortunately, we did not ask participants to report their total time using e-cigarettes within this survey wave. However, given that participants were regular users when first recruited in 2012-2015 and most are still regular users of e-cigarettes in 2022, it is highly likely that most had been using e-cigarettes for at least 7 years. While the views of experienced users are beneficial, these users are also much older than most people who use e-cigarettes, so their views are not representative of all people who use e-cigarettes nationally. Secondly, while we collected detailed opinions from users of e-cigarettes about their feelings regarding FDA regulations via open-ended survey questions, in-depth interviews with these participants would provide a better understanding of their thoughts.

## Conclusions

Common reasons for lack of support for FDA regulation of e-cigarette included concerns of over-regulation, lack of trust in the FDA as a regulatory body, and the FDA’s lack of knowledge on “real world” application of e-cigarettes (i.e. using them as a smoking reduction or cessation tool). To improve trust in the FDA among people who use e-cigarettes, researchers and regulatory bodies should incorporate people who use e-cigarettes perspectives when evaluating e-cigarette regulations. More research should be done on how best to educate smokers about e-cigarettes, and how to correct misinformation about e-cigarettes and their regulation.

## Data Availability

All coded participant statements are available for download at https://scholarsphere.psu.edu/resources/408ce552-bc75-44f0-876f-ad6775e5fdb2.
